# Rapid Screening of Graphitic Carbon Nitrides for Photocatalytic Cofactor Regeneration Using a Drop Reactor

**DOI:** 10.3390/mi8060175

**Published:** 2017-06-02

**Authors:** Xiaowen Huang, Huimin Hao, Yang Liu, Yujiao Zhu, Xuming Zhang

**Affiliations:** 1 Department of Applied Physics, The Hong Kong Polytechnic University, Hong Kong 999077, China; huangxiaowen2013@gmail.com (X.H.); lance2012@mail.dlut.edu.cn (Y.L.); zhuyujiao3@163.com (Y.Z.); 2 The Hong Kong Polytechnic University Shenzhen Research Institute, Shenzhen 518000, China; 3 College of Mechanical Engineering, Taiyuan University of Technology, Taiyuan 030024, China; haohuimin@tyut.edu.cn

**Keywords:** artificial photosynthesis, coenzyme regeneration, graphitic carbon nitride, photocatalysis, photocatalyst screening

## Abstract

Artificial photosynthesis is the imitation of natural photosynthesis, which promises an efficient way to use solar energy to synthesize organic matters, in which the key step is the coenzyme regeneration (NADH/NADPH). To achieve an efficient regeneration rate, various photocatalysts have been developed, such as g-C_3_N_4_ and mesoporous carbon nitride (mpg-C_3_N_4_). Generally, efficiency determination of different photocatalysts requires laborious experiments, high consumption of reagents, and a considerable amount of time. Here, based on the one-step artificial photosystem I method, we processed the analytical experiment in a very simple PDMS well (20 μL, a drop) to achieve a rapid screening of photocatalysts. For comparison, we used two types of graphitic carbon nitrides, few-layer g-C_3_N_4_ and mpg-C_3_N_4_. Compared with the slurry systems, firstly, the regeneration rate of mpg-C_3_N_4_ drop-reactor system is 4.3 times and 7.1 times those of the few-layer g-C_3_N_4_-slurry system and mpg-C_3_N_4_-slurry system, respectively. Secondly, this one-drop method reduces the typical verification time from 90 min to 5 min and lowers the liquid volume from 20 mL to 20 μL. Thirdly, this operation is a pump-free and soft lithography technique-free process. The miniaturization of the photocatalytic reaction in the PDMS well improves the regeneration rates, saves samples, and achieves high-throughput screening of multiple photocatalysts.

## 1. Introduction

**Figure 1:**
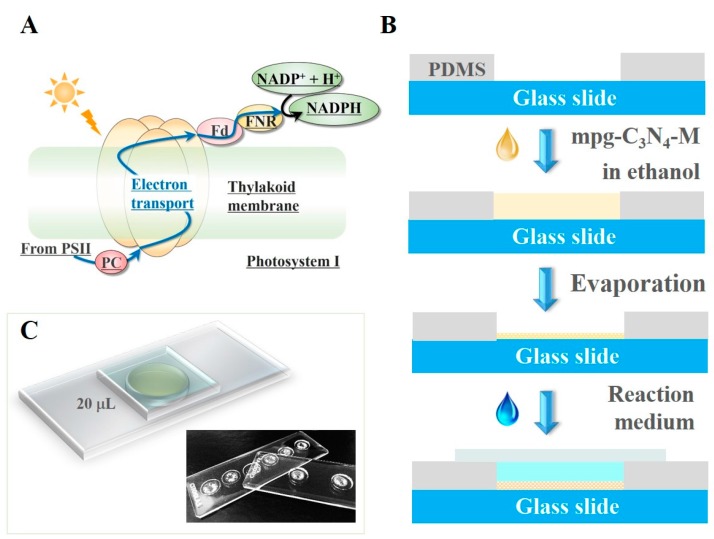
(**A**) Diagram of the electron transport in the Photosystem I reaction center. PSII: photosystem II, PC: plastocyanin, Fd: ferredoxin, FNR: ferredoxin-NADP^+^ reductase. They are the important components on the electron transport chain. (**B**) The schematic illustration of the photocatalytic cofactor regeneration in a drop. (**C**) The schematic and the photos of the fabricated devices.

The discovery of efficient photocatalysts is of vital importance for artificial photosynthesis [1,2,3,4,5,6,7,8,9,10,11,12]. Generally, the efficiency test requires numerous experiments, which consumes large volumes of reagents and takes a long time. This laborious and time consuming nature imposes a technical limit to the quick development of the artificial photosynthesis based coenzyme regeneration. In other fields—for example, water treatment and solar cells—several studies showed their microfluidic methods to achieve the high-throughput screening efficiency. Zhang et al. reported a simple and efficient chip microfluidic chip-based analytical system for rapid screening of photocatalysts [13]. Yuan et al. proposed a high-throughput screening and optimization of binary quantum dot co-sensitized solar cells based on combinatorial chemistry and scanning electrochemical microscopy (SECM) [14]. However, rapid screening has not attracted the due attention to the field of artificial-photosynthesis-based coenzyme regeneration. Here, based on the one-step artificial photosystem I method developed in our previous work [15], we processed the verification experiment on a very simple PDMS well to achieve a rapid coenzyme regeneration, thus for the photocatalysts’ quick screening. The natural photosystem I is an integral membrane protein complex in photosynthesis, as shown in Figure 1A. Correspondingly, the artificial photosystem I method integrated the photocatalysts and the electron mediator (M) in one chip and mimicked the wisdom of photosystem I. The simultaneous assembly of photocatalysts and M could efficiently regenerate NADH from NAD^+^ under visible light irradiation. The whole reaction was processed in a small reactor in the form of a drop, thus, we named this method the ‘drop-reactor’ method.

Carbon nitride, a metal-free efficient photocatalyst, has been proved to be a good photocatalyst material [16,17,18,19,20]. Many works contributed a lot in terms of nanoarchitecture diversity, functionalization of g-C_3_N_4_ by elemental doping or copolymerization [21,22,23,24]. In our previous work [15], the few-layer g-C_3_N_4_ showed a better photocatalytic ability than the bulk g-C_3_N_4_. For further development, this work employed two types of graphitic carbon nitrides, the few-layer g-C_3_N_4_ and mpg-C_3_N_4_. The traditional slurry method was adopted as a comparison. Moreover, this drop-reactor method reduces the typical verification time from 90 min to 5 min and lowers the liquid volume from 20 mL to 20 μL, one-thousandth times of the traditional slurry one. Furthermore, this operation is a pump-free and soft-lithography-free process, and thus needs no microfluidics facilities. Based on these advantages, we propose that the simple, yet highly efficient method offers a convenient tool for photocatalyst screening of artificial photosynthesis.

## 2. Materials and Methods 

### 2.1. PDMS Well Fabrication and Drop-Reactor Method

Polydimethysiloxane (PDMS, DC184) was purchased from Dow Corning Co. (Auburn, MI, USA). We poured the uncross-linked PDMS (PDMS monomers:curing agent = 10:1) on a glass slide (25.4 mm × 76.2 mm), and punched several holes on the PDMS layer by a metal tube (the diameter is 9 mm) after PDMS curing. The well in the PDMS layer is ~0.35 mm thick and 9 mm in diameter. For larger volume, both the height and the diameter of the well could be enlarged by a larger metal tube. 

Pentamethylcyclopentadienylrhodium(III) chloride dimer (CAS Number: 12354-85-7), 2,2′-Bipyridyl (CAS Number 366-18-7), ethanol (CAS Number: 64-17-5), cyanamide (CAS Number: 420-04-2), NH_4_HF_2_ (CAS Number: 1341-49-7) and other chemicals were purchased from Sigma-Aldrich Co. (St. Louis, MO, USA). Ludox-HS 40 colloidal silica suspension (LUDOX^®^ HS-40 colloidal silica 40 wt % suspension in H_2_O, CAS Number 7631-86-9) was also purchased from Sigma-Aldrich Co.

The schematic illustration of the process is shown in Figure 1B as follows: (1) mixing Pentamethylcyclopentadienylrhodium(III) chloride dimer, 2,2′-Bipyridyl and photocatalyst (e.g., mpg-C_3_N_4_) together in ethanol (solvent). The reaction between Pentamethylcyclopentadienylrhodium(III) chloride dimer and 2,2′-Bipyridyl in ethanol formed compound M {[Cp*Rh(bpy)Cl]Cl}, a kind of electron mediator. In this reaction, the electron mediator M {[Cp*Rh(bpy)Cl]Cl} was formed with mpg-C_3_N_4_ as a mixture. (2) removing the ethanol by evaporation at 50 °C. The resulting mixture is the immobilized artificial photosystem I (IAPSI) that contains both photocatalysts and the electron mediator. After the addition of the reaction solution, a thin glass slide was covered on the well to avoid evaporation. 

### 2.2. Synthesis of mpg-C_3_N_4_ and Few-Layer g-C_3_N_4_

The mpg-C_3_N_4_ material was synthesized as follows [25]: 5 g of cyanamide and 12.5 g of Ludox-HS 40 colloidal silica suspension (cyanamide:silica = 1:1 of solid ratio) were mixed until cyanamide was completely dissolved. The oil bath (a part of the rotary evaporator) at 100 °C was used to remove water which took ~12 h. Without water, a white solid (the mixture of the cyanamide and silica) was found. After grounding the obtained white solid in a mortar, we transferred that to a crucible and heated with the lid on under air at 2.3 °C/min up to 550 °C (this step takes for 4 h) and kept at 550 °C for another 4 h. The resultant yellow powder was treated with 4 M NH_4_HF_2_ solution (toxic/corrosive) and stirred for 48 h (Caution needs to be exercised). The dispersion was then filtered; the filtrate was thoroughly rinsed with deionized water and ethanol. After the filtering procedure, the yellow powder was dried under vacuum at 60 °C overnight. Few-layer g-C_3_N_4_ was obtained through the thermal condensation of cyanamide at 550 °C (4 h) under air and used as the control sample.

### 2.3. Material Characterization

The Fourier transform infrared (FTIR) spectrum was collected using a Vertex 70, Bruker Corporation (Madison, WI, USA), FTIR spectrometer. X-ray powder diffraction (XRD) measurements were performed on a D8 Diffractometer from Bruker instruments. The transmission electron microscopy (TEM) images were taken on a TEM grid to perform high-resolution morphology characterization using a JEM2100F TEM (JEOL Ltd., Tokyo, Japan) system operating at 200 kV. The UV–Vis absorbance spectra were recorded using a UV2550 spectrophotometer (Shimadzu Scientific Instruments, Kyoto, Japan). The electron mediator structure was detected by the nuclear magnetic resonance (NMR) analysis (300 MHz, Bruker Corporation).

### 2.4. NADH Photoregeneration

Photoregeneration of NADH was carried out by filling the PDMS well with 20 μL reaction medium, irradiated with a xenon lamp (300 W) through a 420-nm cut-off filter, avoiding the UV light-induced damage of the enzyme. The reaction medium (pH 8.0) was composed of NAD^+^ (0.2 mM), TEOA (15 *w*/*v* %), and phosphate buffer (100 mM); 20 μL of the reaction medium was filled into the prepared chip. The immobilized g-C_3_N_4_ was 20 μg in each chip and the complex M was equal to 0.25 mM. During the reaction, we used a fan to cool down the devices. Thus, within several minutes, the temperature of the reaction systems was controlled well at the room temperature.

## 3. Results

### 3.1. PDMS Well Fabrication and Drop-Reactor Method

The fabricated device is shown in Figure 1C. The parallel wells can be made on the same chip. This allows for screening various catalyst samples at the same time and thus increasing the throughput of the screen. In the following experiments, we used the 0.35-mm-thick wells, each of which could accommodate a drop of reaction liquid up to 20 μL. In this sense, each reaction occurred inside a drop. 

### 3.2. Characterizations of mpg-C_3_N_4_ and Few-Layer g-C_3_N_4_

**Figure 2:**
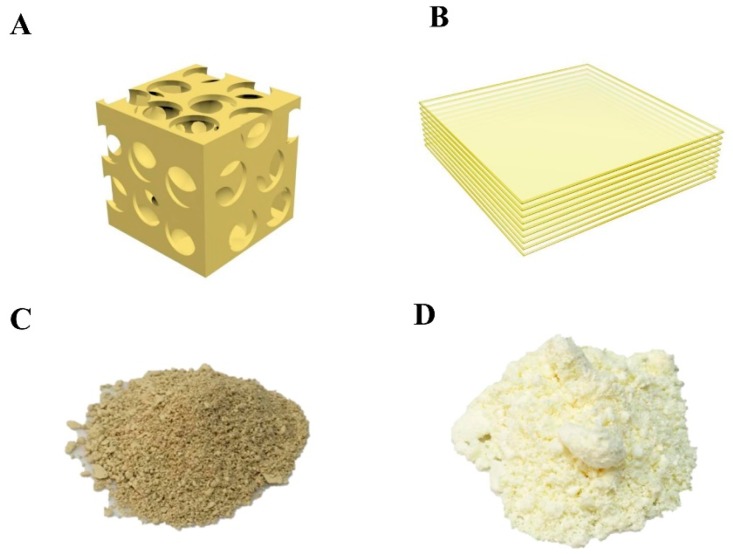
(**A**) The schematic structure of mpg-C_3_N_4_ which contains numerous mesopores because of silica nanoparticle templates. (**B**) The few-layer g-C_3_N_4_ is the typical layer-by-layer structure, without the mesopores in mpg-C_3_N_4_. (**C**,**D**) Photos of mpg-C_3_N_4_ and the few-layer g-C_3_N_4_, the color of mpg-C_3_N_4_ is much darker than the few-layer g-C_3_N_4_, inferring its better visible light absorption.

The prepared mpg-C_3_N_4_ and few-layer g-C_3_N_4_ had several differences. First, mpg-C_3_N_4_ was made with the silica nanoparticles as templates, thus it contained numerous mesopores after dissolving the silica nanoparticles (Figure 2A). The few-layer g-C_3_N_4_ was the typical layer-by-layer structure, without the mesopores, as shown in Figure 2B. Second, the color of mpg-C_3_N_4_ (Figure 2C) is much darker than the few-layer g-C_3_N_4_ (Figure 2D), showing the better visible light absorption of mpg-C_3_N_4_. Third, the mpg-C_3_N_4_ had a higher mesopore surface area (ca. 200 m^2^/g) as well as more active sites for interfacial photoreactions [26].

**Figure 3:**
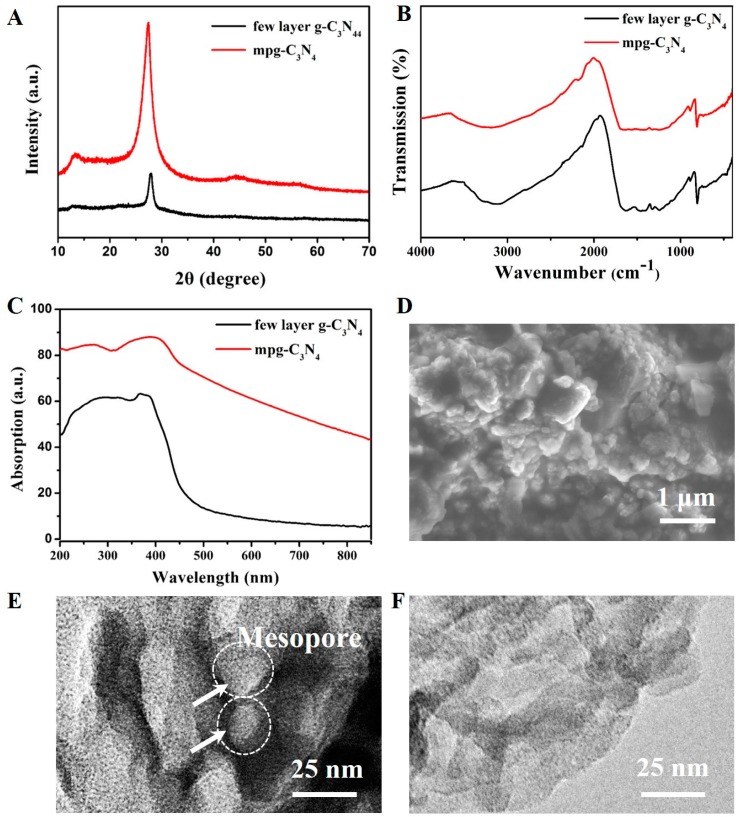
Characterization of mpg-C_3_N_4_ and few-layer g-C_3_N_4_. (**A**) X-ray powder diffraction (XRD) spectra of mpg-C_3_N_4_ and few-layer g-C_3_N_4_ with two peaks at 12.7° and 27.8°, the typical characteristic peaks for g-C_3_N_4_. (**B**) Fourier transform infrared (FTIR) spectra of mpg-C_3_N_4_ and few-layer g-C_3_N_4_, showing typical C-N heterocycle stretches at 1251, 1325, 1419, 1571, and 1639 cm^−1^, as well as the characteristic breathing mode of triazine units at 810 cm^−1^. (**C**) UV–Vis absorption spectra of mpg-C_3_N_4_ and few-layer g-C_3_N_4_. The mpg-C_3_N_4_ show better visible light absorption. (**D**) The scanning electron microscopy (SEM) image of mpg-C_3_N_4_. (**E**) The transmission electron microscopy (TEM) image of mpg-C_3_N_4_, showing the random mesopores. (**F**) The TEM image of the few-layer g-C_3_N_4_ material, showing no mesopores.

Figure 3A shows the XRD spectrum of the mpg-C_3_N_4_ and few-layer g-C_3_N_4_. The two XRD peaks at 12.7°and 27.8° show the lattice planes parallel to the c-axis and the stacking of the conjugated aromatic system, respectively. Figure 3B shows the FTIR broad peaks between 3000 and 3500 cm^−1^ are attributed to the N-H band. The peaks at 1251, 1325, 1419, 1571, and 1639 cm^−1^ correspond to the typical stretching modes of C-N heterocycles. The peak at 810 cm^−1^ corresponds to the characteristic breathing mode of triazine units, which is compliant with the reported data [27]. The UV–Vis absorption spectrum of the few-layer g-C_3_N_4_ is broad, ranging from UV light to visible light (see Figure 3C), the visible light absorption ability of mpg-C_3_N_4_ is stronger than the few-layer g-C_3_N_4_. From the scanning electron microscopy (SEM, Bruker Corporation) image (Figure 3D), we can see the mpg-C_3_N_4_ has a mesoporous structure. The TEM images in Figure 3E,F clearly show that the mpg-C_3_N_4_ has random mesopores whereas the few-layer g-C_3_N_4_ only has a graphene-like structure.

### 3.3. NADH Photoregeneration

The final products were shown in Figure 1C. The parallel wells can be made in the same chip. This allows for screening various catalyst samples at the same time and thus increases the throughput of screen. In the following experiments, we used the 0.35-mm-thick wells, each of which could accommodate a drop of reaction liquid up to 20 μL. In this sense, the reaction occurred in a drop.

**Figure 4:**
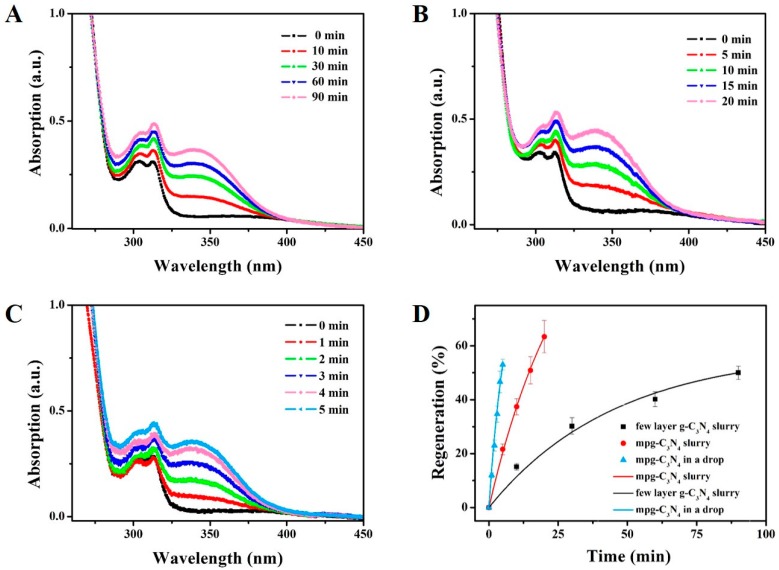
Experimental results of the NADH regeneration rates in three systems, (**A**) The few-layer g-C_3_N_4_-slurry system, (**B**) The mpg-C_3_N_4_-slurry system, and (**C**) The mpg-C_3_N_4_ drop-reactor system. (**D**) Comparison of the regeneration rates of the three systems. The regeneration in the drop reactor is ~4.3 times and ~7.1 times faster than the other two in terms of NADH regeneration, showing its potential as a rapid photocatalyst detection platform.

We detected the NADH regeneration in three systems: the few-layer g-C_3_N_4_-slurry system, the mpg-C_3_N_4_-slurry system and the mpg-C_3_N_4_ drop-reactor system. The former two are used as the comparison. The distance between the reactor system and the xenon lamp was fixed at 10 cm. Before the illumination, the adsorption–desorption equilibrium was achieved. The concentration of NADH was calculated by measuring the absorption of the diluted reaction system at 340 nm, on which the extinction coefficient was 6220 M^−1^·cm^−1^. Figure 4A shows the UV–Vis spectrum changes of the few-layer g-C_3_N_4_-slurry system within 90 min, at which time the regeneration is 50%. Figure 4B plots the data of the mpg-C_3_N_4_-slurry system, in which it only takes 15 min to achieve 50.9% and 20 min to get 63.4%. Thus, the mpg-C_3_N_4_ shows better catalytic ability than the few-layer g-C_3_N_4_, both are in the slurry system.

### 3.4. Reaction Mechanism of the Two Systems

The small drop-reactor system functions better than the traditional one in the coenzyme photo-regeneration. In principle, semiconductor photocatalysts (g-C_3_N_4_ and mpg-C_3_N_4_) absorbs the appropriate photon (*hν ≥ E*_0_, *E*_0_ is the bandgap of the semiconductor photocatalyst) to excite an electron in the conduction band, leaving a hole in the valence band. The electron moves to the surface-active sites (surface reduction cite) for surface redox reactions [28].

Considering the reaction mechanism, the drop-reactor system brings some merits to this photo-regeneration. First, the surface area to volume ratio in the drop-reactor system is larger than the traditional slurry system. This merit increases the chance of the photoreaction. Second, the light irradiation in the drop-reactor system is more uniform than the slurry system. Second, the light irradiation in the drop-reactor system is more uniform than the slurry system. With a thin photocatalyst film and a thin layer of fluid, the irradiation in the transparent, sealed, and small drop-reactor system is almost uniform, utilizing the abundant photons and thus having a high photon efficiency. However, in the slurry system, only the surface parts of the photocatalysts get the abundant photon irradiation. The photocatalysts away from the light source show low photon usage. Third, the small drop-reactor system has the shorter diffusion length. The small volume makes it easier for the substrate to contact the reaction site on the photocatalysts.

## 4. Discussion

This improvement can be attributed to its mesoporous structure, which offers more reactive sites and improves the inner scattering induced-visible light absorption that leads to more photo-generated charge carriers. The slurry system can be used to characterize the photocatalysts. However, the verification time is too long and the reagent consumption is still large. Systems with shorter time requirement and lower reagent consumption will be more useful.

These requirements are well satisfied by the mpg-C_3_N_4_ drop-reactor system. As shown in Figure 4C, the absorption at 340 nm rises to 11.9% at 1 min and 53% at 5 min. In Figure 4D, we show a direct comparison between these three systems, where the regeneration percentage is plotted as a function of time with the exponential relationship

*y* = *A* exp(−*x*/*t_c_*) + *y*_0_ (1)

where *t_c_* is the characteristic chemical time, defined as the time required for the concentration of *A* to fall from its initial value to a value equal to 1/e times of the initial value.

After fitting the data in origin, we found the characteristic chemical time is ~6.4 s, 27.4 s and 45.7 s, respectively. Thus, the regeneration rate of mpg-C_3_N_4_ drop-reactor system is ~4.3 times and 7.1 times of those of the few-layer g-C_3_N_4_-slurry system and the mpg-C_3_N_4_-slurry system, respectively.

## 5. Conclusions

We proposed a rapid screening of the photocatalyst method using a drop reactor, which is advantageous over the slurry method in terms of fast detection, simple process, and low reagent consumption. This shows its potential for multiple photocatalyst screening in the field of artificial photosynthesis.
